# Ferric citrate controls serum phosphorus in dialysis patients: retrospective data

**DOI:** 10.5414/CN109057

**Published:** 2017-05-31

**Authors:** Debra J. Hain, Meredith Marinaro, David W. Koeper, Melissa A. Rosenthal, Salvatore Chillemi, Jennifer M. Huffman, Teresa Gerbeling, James M. Pritsiolas, Lisa C. Loram, Pablo E. Pergola

**Affiliations:** 1 Florida Atlantic University, Boca Raton, and Department of Nephrology Cleveland Clinic Florida, Weston, FL,; 2 University of Connecticut, Farmington, CT,; 3 Fox Valley Nephrology, Neenah, WI,; 4 Los Alamitos HD Clinic, Los Alamitos, CA,; 5 North Georgia Kidney Specialists, Marietta, GA,; 6 Kidney Associates of Kansas City, Kansas City, MO,; 7 Dialysis Center of Lincoln, Inc., Lincoln, NE,; 8 CarePoint Health Medical Group, Bayonne, NJ,; 9 Keryx Biopharmaceuticals, Inc., and; 10 Renal Associates PA, San Antonio, TX, USA

**Keywords:** phosphate binder, peritoneal dialysis, hemodialysis, hyperphosphatemia, anemia

## Abstract

Ferric citrate is an approved phosphate binder for use in patients with chronic kidney disease on dialysis. Clinical trials demonstrated that ferric citrate controlled serum phosphorus levels and increased iron stores. The aim of this retrospective chart review was to evaluate real-world bone mineral and anemia parameter data from patients treated with ferric citrate. 92 adult dialysis patients taking ferric citrate (average starting dose of 6 tablets/day) for at least 6 months were included. Bone mineral, anemia, and iron biomarker levels were extracted from patient medical records before and during the first 6 months of ferric citrate treatment; 21 (23%) patients were phosphate binder naïve, and 71 (77%) patients had been on other phosphate binders. Before starting ferric citrate, 22% of patients had serum phosphorus ≤ 5.5 mg/dL, increasing to 65% of patients at 6 months of treatment (month 6). Mean (standard error of the mean (SEM)) baseline serum phosphorus was 6.55 ± 0.17 mg/dL decreasing to 5.40 ± 0.17 mg/dL at month 6. Mean (SEM) baseline hemoglobin, ferritin, and transferrin saturation were 10.6 ± 0.2 g/dL, 734 ± 65 ng/mL, and 27.1 ± 1.6%, respectively, and 11.1 ± 0.2 g/dL, 947 ± 66 ng/mL, and 37 ± 1.9%, respectively, at month 6. The serum phosphorus and anemia biomarker levels observed in this retrospective chart review were similar to those seen in clinical trials.

## Introduction

Hyperphosphatemia is a common problem among adults with chronic kidney disease (CKD) on dialysis that, without intervention, has been associated with increased morbidity and mortality [1]. As a result, the use of safe and effective phosphate binders has been an essential component of treatment plans for CKD patients on dialysis to reduce the amount of phosphorus absorbed from the diet and, thus, reduce serum phosphorus levels. 

Ferric citrate (Auryxia^®^, Boston, MA, USA) was approved in the United States (US) in 2014 for the control of serum phosphorus levels in patients with CKD on dialysis [2]. The long-term safety and efficacy of ferric citrate in CKD patients on dialysis was studied in a phase 3 clinical trial with a 52-week, randomized, active-controlled period comparing ferric citrate to sevelamer carbonate and/or calcium acetate, followed by a 4-week randomized, placebo-controlled period (NCT01191255) [3]. In this study, ferric citrate reduced and maintained serum phosphorus levels similarly to the active control and significantly better than placebo and had a similar safety profile to that of the active control group. 

Given the dynamic change of treatment practices for patients on dialysis in the US, there is a need to understand how ferric citrate is used in real-world practice and how outcomes of patients taking ferric citrate compare to the published clinical trial data. 

## Materials and methods 

A retrospective chart review was performed by 7 clinicians at clinics across the US. Clinicians included community healthcare professionals (e.g., nephrologists, dietitians) who routinely treat patients with CKD on dialysis. To be included in the study, patients on dialysis were required to have been prescribed ferric citrate (alone or in combination with another binder) for the control of serum phosphorus, based on the provider’s clinical decision, for a minimum of 6 months. Clinicians reviewed the charts from their practices and selected patients who met the inclusion criteria (decision was at the clinical level, not the facility level). 

Data were collected and captured from routine clinical care that was documented in patient medical records. Laboratory values were not collected every month for all parameters but rather as directed by routine clinical care (i.e., intact parathyroid hormone (iPTH), transferrin saturation (TSAT)). 

Demographic and baseline health data included gender, age, race, baseline weight, comorbidities (diabetes, hypertension), dialysis status (time since initiation, type of dialysis), and previous phosphate binder information (type and number of pills/day). Clinicians collected data from patient medical records regarding treatment with ferric citrate, including duration of treatment, average pill count per day for each month of treatment, and concomitant use of additional phosphate binders. Laboratory data were collected following initiation of treatment with ferric citrate at baseline (month 0) and monthly, if available, which included serum phosphorus, serum calcium, and iPTH. Serum ferritin, TSAT, and hemoglobin (Hgb), as well as intravenous (IV) iron dosing, were collected for 3 months before starting ferric citrate treatment and monthly following initiation of treatment with ferric citrate. All available laboratory data from patient charts for these parameters are included in the analysis. 

The clinicians determined whether any of the collected laboratory data were considered to be adverse events or serious adverse events using Kidney Disease Improving Global Outcomes (KDIGO) targets as reference ranges for ferritin, TSAT, and Hgb, and the National Kidney Foundation’s Kidney Disease Outcomes Quality Initiative (KDOQI) targets as reference ranges for phosphorus, calcium, and iPTH [4, 5, 6]. Descriptive data are presented, and laboratory values are presented in the text as mean ± standard error of the mean (SEM). 

## Results 

The demographics and baseline characteristics of the 92 patients are presented in Table 1. The majority were male (63%) and Caucasian (52%). The mean time on dialysis was 38 months with a range of 0 – 204 months, indicating that both incident and prevalent dialysis patients were included in the study. The distribution of types of dialysis of patients in the study included 67% of patients that received in-center hemodialysis (HD), 27% on peritoneal dialysis (PD), and 5% that received home HD. Data for home HD and in-center HD patients are presented together. Most patients (77%) had previously used a phosphate binder before switching to ferric citrate. Patients that were phosphate binder naïve before initiating ferric citrate (binder-naïve subgroup) had been on dialysis for an average of 6 months compared to 48 months for those patients that switched from a different phosphate binder (binder-experienced subgroup). 

### Phosphate binder use and pill burden 

Average daily prescribed pill count was collected for phosphate binders taken before starting ferric citrate treatment and, if concomitant binders were taken, during ferric citrate treatment. 23% of patients had not previously been treated with a phosphate binder. Among the 71 patients in the binder-experienced subgroup, 52% were on sevelamer only, and their average pill count was 11 pills/day. For the 28% of patients in the binder-experienced subgroup that received a calcium-based binder, their average pill count was 9 pills/day. Those on combination of sevelamer and calcium-based binders (14%) averaged 16 pills/day (Table 2). 

### Ferric citrate use 

The mean starting dose of ferric citrate was 6 pills/day, which increased to 7 pills/day at months 3 and 6. The type of dialysis a patient received did not influence the mean starting dose of ferric citrate; both the HD and PD subgroups started at 6 pills/day. The binder-naïve subgroup started at a mean dose of 5 pills/day vs. 6 pills/day for the binder-experienced subgroup. Following 6 months of ferric citrate treatment, the average pill count for the HD subgroup was 7 pills/day vs. 6 pills/day for the PD subgroup. At 6 months of treatment, the average pill count for the binder-naïve subgroup was 6 pills/day vs. 7 pills/day for the binder-experienced subgroup. 17 (18%) patients began ferric citrate treatment in addition to continuing to take a previously prescribed phosphate binder. 

### Mineral bone biomarkers 

**Figure 1.**
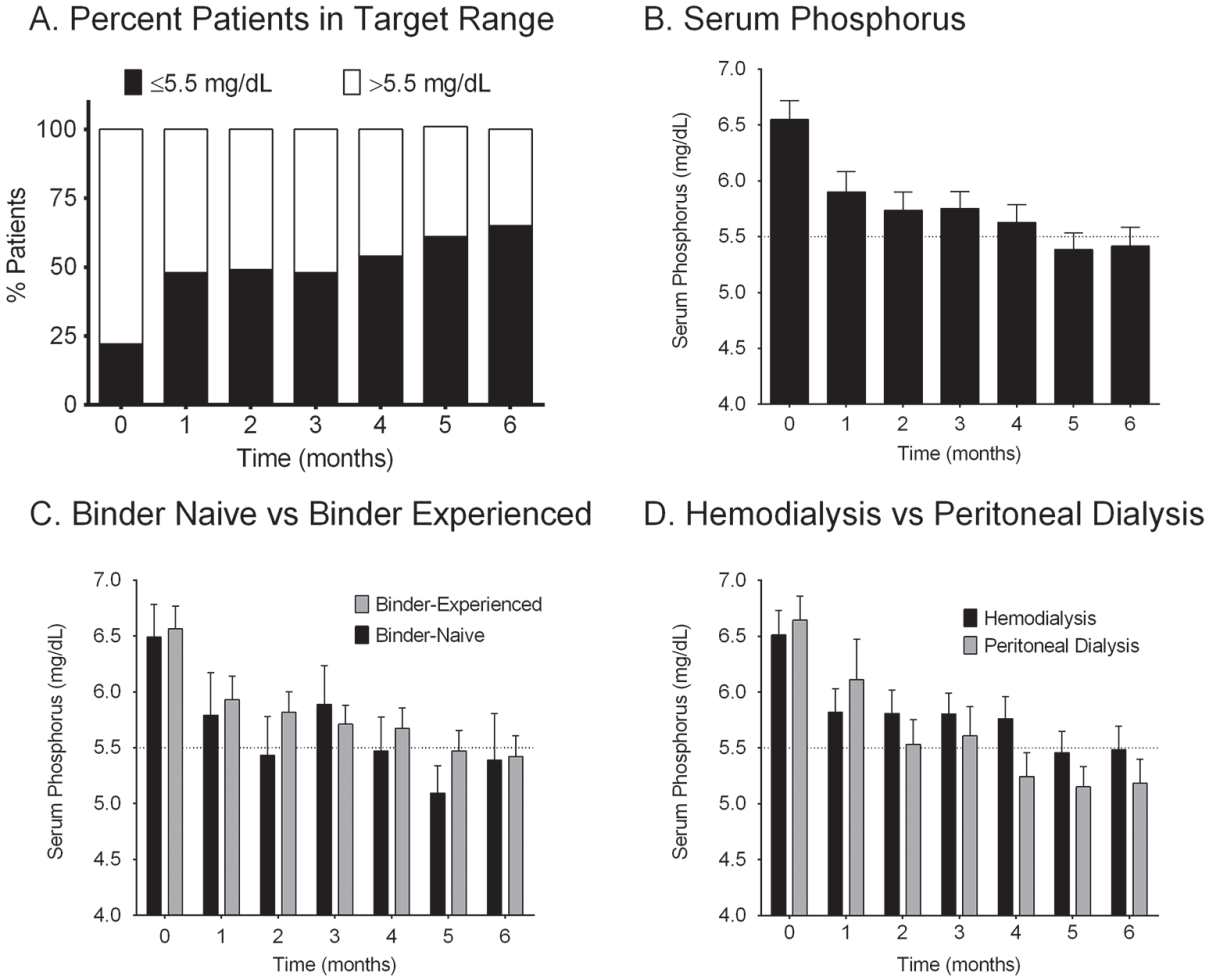
Serum phosphorus levels overall and by subgroup. Data presented are mean ± standard error of the mean (n = 92). A: Percent of patients within KDOQI target serum phosphorus range of ≤ 5.5 mg/dL (n = 92 at month 0 and n = 81 at month 6); B: Serum phosphorus levels over time for all patients (n = 92 at month 0 and n = 81 at month 6); C: Serum phosphorus levels for binder experience subgroups (binder-experienced subgroup: n = 71 at month 0 and n = 64 at month 6; binder-naïve subgroup n = 21 at month 0 and n = 17 at month 6); D: Serum phosphorus levels for dialysis subgroups (HD: n = 67 at month 0 and n = 62 at month 6; PD: n = 25 at month 0 and n = 19 at month 6).

Mineral bone biomarkers collected included serum phosphorus, serum calcium, and iPTH. Figure 1A shows the percentage of patients within the KDOQI target range of ≤ 5.5 mg/dL for serum phosphorus before and during ferric citrate treatment. Before starting treatment, 22% of patients were within the serum phosphorus target range, while 48% and 65% of patients were within the target range at months 1 and 6, respectively. For all patients, mean serum phosphorus levels decreased from 6.55 ± 0.17 mg/dL before starting ferric citrate to 5.90 ± 0.18 mg/dL at month 1 and 5.41 ± 0.17 mg/dL at month 6 (Figure 1B) (Table 3). The binder-naïve subgroup had slightly lower baseline mean serum phosphorus (6.49 ± 0.29 mg/dL) compared to the binder-experienced subgroup (6.56 ± 0.20 mg/dL); however, both subgroups showed a similar decrease and maintenance of serum phosphorus from months 1 through 6 (Figure 1C). Patients in the PD and HD subgroups had similar baseline mean serum phosphorus levels (6.64 ± 0.22 mg/dL and 6.51 ± 0.22 mg/dL, respectively), but the PD subgroup had lower mean serum phosphorus beginning at month 2, which was maintained through month 6 (PD subgroup: 5.18 ± 0.22 mg/dL; HD subgroup: 5.49 ± 0.21 mg/dL) (Figure 1D). Mean serum calcium and iPTH levels were both maintained from baseline through month 6 for all patients (Table 3). 

### Iron biomarkers 

**Figure 2.**
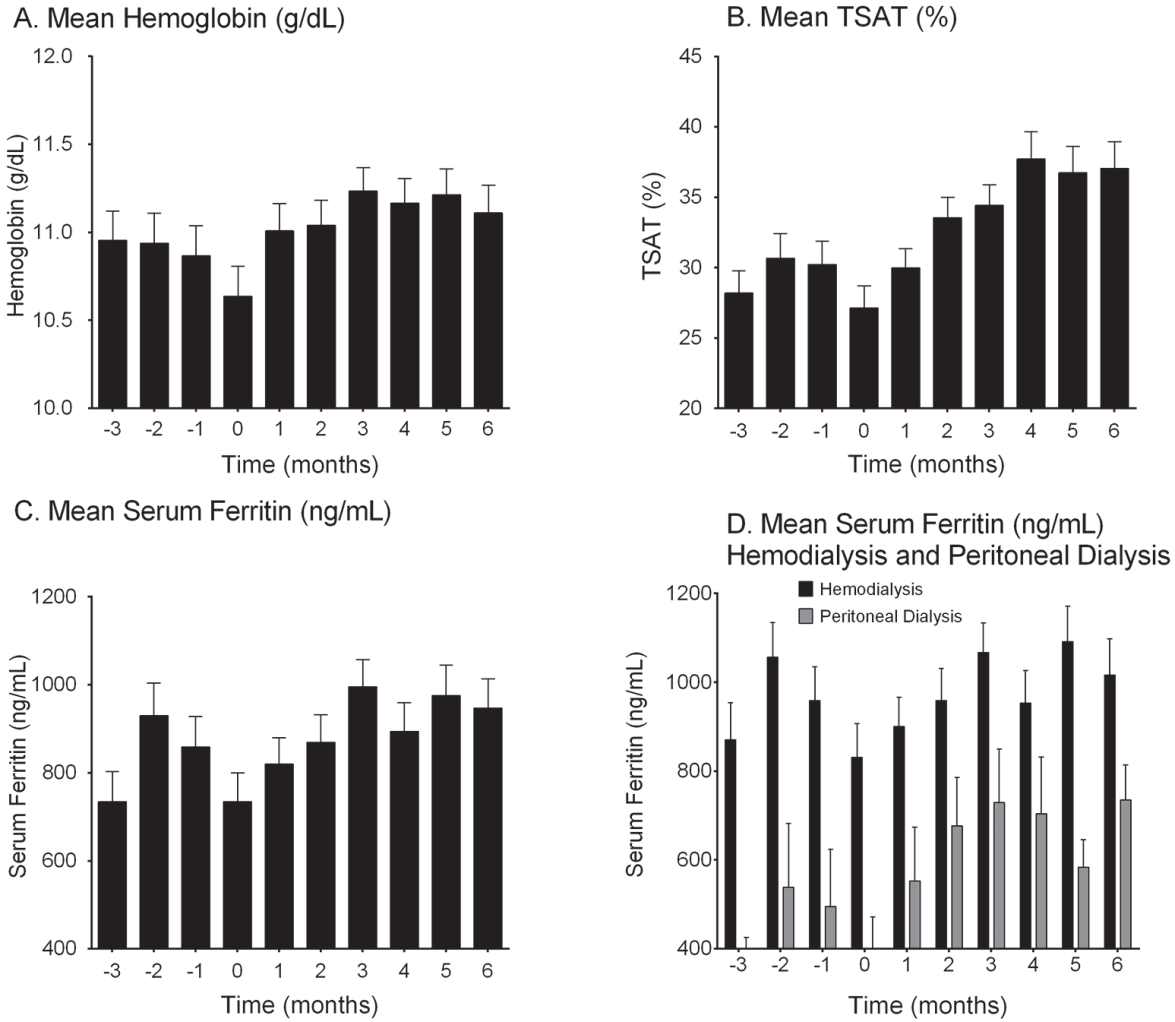
Mean (standard error of the mean) iron biomarker measurements over time. Data presented are mean ± standard error of the mean (n = 92). A: Mean hemoglobin for all patients (n = 76 at month –3 and n = 76 at month 6); B: Mean TSAT for all patients (n = 62 at month –3 and n = 70 at month 6); C: Mean serum ferritin for all patients (n = 59 at month –3 and n = 65 at month 6); D: Mean serum ferritin for dialysis subgroups (HD subgroup: n = 43 at month –3 and n = 49 at month 6; PD subgroup: n = 16 at month –3 and n = 16 at month 6).

Measurements of iron included Hgb (g/dL), TSAT (%), and serum ferritin (ng/mL) and are presented from 3 months before starting ferric citrate treatment through month 6 after starting ferric citrate treatment in Figure 2. Iron biomarker levels increased from baseline within the first 3 months of treatment, and these levels were maintained through month 6. 

There was high variability in both the values and availability of iron biomarker levels prior to initiating treatment with ferric citrate. Baseline values (month 0) for Hgb, TSAT, and ferritin were 10.6 ± 0.2 g/dL, 27.1 ± 1.6%, and 734 ± 65 ng/mL, respectively. Following 3 months of ferric citrate treatment, mean Hgb was 11.2 ± 0.1 g/dL, which was maintained through month 6 (11.1 ± 0.2 g/dL). Similarly, mean TSAT and serum ferritin were 34.4 ± 1.5% and 995 ± 61 ng/mL, respectively, at month 3 and 37.0 ± 1.9% and 947 ± 66 ng/mL, respectively, at month 6. 

The PD subgroup had similar mean Hgb and TSAT both at baseline (10.8 ± 0.3 g/dL and 27.9 ± 2.0%, respectively) and throughout the study (11.4 ± 0.3 g/dL and 37.8 ± 2.5%, respectively, at month 6) compared to those in the HD subgroup (10.6 ± 0.2 g/dL and 24.9 ± 1.8% at baseline, and 11.1 ± 0.2 g/dL and 35.1 ± 2.1% at month 6). Mean ferritin was lower in the PD subgroup (401 ± 70 ng/mL at baseline and 735 ± 78 ng/mL at month 6) compared to the HD subgroup (831 ± 76 ng/mL at baseline and 1,016 ± 81 ng/mL at month 6) (Figure 2D). 

The binder-naïve subgroup had lower mean Hgb at baseline (9.9 ± 0.3 g/dL) compared to the binder-experienced subgroup (10.9 ± 0.2 g/dL), but similar mean Hgb levels were observed between subgroups following 6 months of treatment with ferric citrate (10.9 ± 0.3 g/dL for the binder-naïve subgroup, 11.2 ± 0.2 g/dL for the binder-experienced subgroup, at month 6). The binder-naïve subgroup had higher mean TSAT at baseline (28.8 ± 1.8%) compared to the binder-experienced subgroup (22.2 ± 3.1%), but similar mean TSAT levels were observed between subgroups following 6 months of treatment with ferric citrate (36.8 ± 2.3% for the binder-naïve subgroup, 37.8 ± 3.1% for the binder-experienced subgroup). Similarly, the binder-naïve subgroup had a lower mean baseline serum ferritin (414 ± 94 ng/mL) compared to the binder-experienced subgroup (857 ± 75 ng/mL), but the two subgroups had similar levels at the end of 6 months of treatment (905 ± 154 ng/mL for the binder-naïve subgroup, 959 ± 73 ng/mL for the binder-experienced subgroup). 

### Intravenous iron use 

IV iron monthly dosing was collected during the same timeframe as the laboratory data. The number of patients that were prescribed IV iron and the amount of IV iron administered per patient was highly variable over time. Participating clinicians followed their own clinic-specific protocols for IV iron and erythropoiesis-stimulating agent (ESA) dosage and use with varying formulations and dosing schedules (reflective of real-world treatment). Overall, 26 patients were prescribed IV iron treatment 3 months before starting ferric citrate, 43 patients at the time of starting ferric citrate, and 35 patients after 6 months of ferric citrate treatment. 

### Safety 

Using KDIGO reference ranges, 392 laboratory values that were outside of the target ranges were identified in 83 patients [3, 5]. None of the out-of-range laboratory values was assessed by clinicians to be adverse events or serious adverse events. There were 3 serious adverse events reported spontaneously during data collection (gastrointestinal hemorrhage, renal transplant, and *E. coli* sepsis with liver abscess), but none were suspected to be drug related, as identified by the clinicians. Five patients stopped ferric citrate after 3 months of treatment. Three of these patients received kidney transplants, 1 discontinued dialysis, and 1 was lost to follow-up. 

## Discussion 

In this retrospective analysis of CKD patients on dialysis treated with ferric citrate in real-world practice settings, ferric citrate was shown to reduce and maintain serum phosphorus levels over the 6 months of the analysis, with no changes in serum calcium and iPTH levels. Following only 1 month of treatment with ferric citrate, the number of patients within the target serum phosphorus range doubled (23% vs. 48%), and nearly 2/3 of the patients studied were within the target range by month 6. The effect of ferric citrate on serum phosphorus levels was similar regardless of whether a patient was phosphate binder naïve before initiating ferric citrate treatment (binder-naïve subgroup) or had switched from a different phosphate binder (binder-experienced subgroup). However, following 2 months of treatment with ferric citrate, overall lower mean serum phosphorus values were observed among patients receiving PD compared to patients receiving HD. 

Although not specifically indicated for the maintenance of iron levels, ferric citrate has been shown to increase iron stores in CKD patients [2, 7]. Therefore, we analyzed the effect of ferric citrate on biomarkers of iron (Hgb, TSAT, and serum ferritin). These iron biomarker measurements indicated an increase of iron stores over time that was maintained through month 6. As with the serum phosphorus values, a difference was observed in the iron biomarkers between patients receiving PD vs. patients receiving HD. Patients receiving PD had similar Hgb values at baseline and throughout the 6-month treatment period compared to patients receiving HD; however, mean ferritin levels were higher for the HD subgroup. Those patients who were not on a phosphate binder before initiating ferric citrate had lower iron biomarker levels compared to patients who previously took a phosphate binder, but these iron indicator levels were similar across the two subgroups following 6 months of ferric citrate treatment. 

Data from this analysis were similar to the data observed in the phase 3 trial and reported in the ferric citrate (Auryxia^®^) label [2, 3]. Following the 52-week, active-controlled period of the phase 3 study, patients randomized to ferric citrate had a mean serum phosphorus measurement of 5.36 mg/dL, which was similar to the mean observed in our study of 5.42 mg/dL after 6 months of treatment. Measurements of iron stores were also similar, including mean Hgb, ferritin, and TSAT values between the phase 3 trial (11.7 g/dL, 899 ng/mL, and 39%, respectively) and our analysis (11.1 g/dL, 947 ng/mL, and 37%, respectively, at month 6). 

In addition, data from this analysis were consistent with national sampling in the US, based on the Dialysis Outcomes & Practice Patterns Study Program (DOPPS) database. The DOPPS Practice Monitor tracks and reports clinical data on HD practices in the US over time using a sample of more than 11,000 patients from more than 200 HD facilities. From 2010 – 2015, 62 – 72% of patients tracked in the DOPPS Practice Monitor had serum phosphorus levels within the target range of ≤ 5.5 mg/dL [8]. By month 6 of our study, 65% patients were within the target phosphorus range. The slightly lower number on target may be related to patient selection for this analysis. It is likely that most patients were switched because their previous binder was not effective or was causing side effects (only 22% of patients were within the target serum phosphorus level at baseline), but some patients may have had persistently elevated phosphorus resulting from poor compliance with binders or poor diet that may have persisted after the treatment switch. 

In regards to anemia biomarkers, from 2010 – 2015, the DOPPS Practice Monitor reported that ~ 50 – 60% of patients in the national sample had TSAT values ≤ 29%, 55 – 70% had serum ferritin values of ≤ 799 ng/mL, and 41 – 72% had Hgb values ≥ 11.0 g/dL. Interestingly, these reported values are similar to the mean values observed in our study. The IV iron dosing in this data set was highly variable and thus difficult to interpret. However, the phase 3 clinical trial in end-stage renal disease subjects demonstrated a reduction in both IV iron and ESA use over the 52-week period [3, 7]. 

Although there are limitations to this study, having real-world data can provide valuable clinical information for prescribing clinicians. The following presents limitations of the study: (1) The sample size in this study is relatively small (92 patients), yet this is the largest real-world data set for patients on ferric citrate to date. (2) Patients were only included in the analysis if they had been taking ferric citrate for a minimum of 3 months (6 months of data were collected). As a result, patients that could not tolerate ferric citrate or those that discontinued treatment with ferric citrate for any other reason were excluded. Despite this limitation, there was little apparent selection bias in this study, as there were few inclusion criteria. (3) A combination of both incident and prevalent dialysis patients was included; however, the overall population studied provides a representation of the diversity of patients receiving ferric citrate in current practice. (4) As expected from a retrospective chart review, data collected are limited to the available data from the patients’ medical records. Pill count for both ferric citrate and other phosphate binders used previously or concomitantly were based on the prescribed pill counts obtained from the medical records, and no specific measures of adherence were used. However, post-hoc analysis of the pivotal phase 3 clinical trial of ferric citrate vs. an active control revealed an average of 81% patient adherence to both the study drug and active control [9]; thus, it is reasonable to assume similar adherence rates in this study. (5) Limited data on prescribed ESA and IV iron use and dosage may be due to alterations in the ESA administration protocols over the 6-month period studied in some of the participating clinics, as well as different clinic-specific protocols for ESA prescription and use. A future study of both ESA and IV iron use among ferric citrate patients may be valuable. (6) Finally, although our safety data are limited to reported laboratory values, no serious adverse events were reported. 

In conclusion, real-world data from this analysis have demonstrated that ferric citrate is an effective phosphate binder with 65% of the patients within the target phosphorus range and overall increased iron stores and maintenance of Hgb levels following 6 months of treatment. No new safety signal was identified in this study. 

## Acknowledgments 

The authors acknowledge Keryx Biopharmaceuticals, Inc. (Boston, MA, USA) for supporting the preparation of this manuscript and Lisa Ambrosini Vadola, PhD, of Whitsell Innovations, Inc. (Chapel Hill, NC, USA) for her assistance in the preparation of this manuscript. 

## Conflict of interest 

This study was supported by Keryx Biopharmaceuticals, Inc. Dr. Hain has received speaker’s fees from Amgen (Thousand Oaks, CA, USA) and was on the advisory board for ZS Pharma (San Mateo, CA, USA). Ms. Marinaro has received speaker’s fees from Keryx Biopharmaceuticals, Inc. Dr. Koeper has received speaker’s fees from Keryx Biopharmaceuticals, Inc. and Amgen. Dr. Rosenthal has received speaker’s fees from Keryx Biopharmaceuticals, Inc. Dr. Chillemi has received speaker’s fees from Keryx Biopharmaceuticals, Inc. Ms. Huffman has received speaker’s fees from Keryx Biopharmaceuticals, Inc. and Mallinckrodt Pharmaceuticals (Staines-Upon-Thames, Surrey, UK). Ms. Gerbeling has received speaker’s fees from Keryx Biopharmaceuticals, Inc. Dr. Lisa Loram is an employee of Keryx Pharmaceuticals, Inc. Dr. Pritsiolas has received speaker’s fees from Keryx Biopharmaceuticals, Inc., Relypsa (Redwook City, CA, USA), Alexion Pharmaceuticals, Inc. (New Haven, CT, USA), and Otsuka America Pharmaceutical, Inc. (Rockville, MD, USA) and has received research funding from Amgen and Luitpold Pharmaceuticals (Shirley, NY, USA). Dr. Pergola has received speaker’s fees from Keryx Biopharmaceuticals, Inc., Sandoz (Holzkirchen, Germany), ZS Pharma, Relypsa, and Viphor Pharma (Glattbrugg, Switzerland). 

**Table 1.** Demographics and baseline characteristics.

Gender, n (%)	
Male	58 (63)
Female	34 (37)
Age (years), mean (range)	57 (25 – 91)
Race, n (%)	
Caucasian	48 (52)
African-American	21 (23)
Asian	4 (4)
Other	11 (12)
Weight (kg), mean (range)	82 (49 – 162)
Diabetes, n (%)	
Insulin-dependent	17 (18)
Non-insulin-dependent	20 (22)
Hypertension, n (%)	84 (91)
Previous phosphate binder, n (%)	
Binder-naïve	21 (23)
Binder-experienced^a^	71 (77)
Type of dialysis, n (%)	
In-center hemodialysis	62 (67)
Home hemodialysis	5 (5)
Peritoneal dialysis	25 (27)
Time on dialysis (months), mean (range)	
By type of dialysis	
All dialysis patients	38 (0 – 204)
In-center hemodialysis	45 (0 – 204)
Home hemodialysis	25 (3 – 25)
Peritoneal dialysis	16 (0 – 108)
By previous binder use	
Binder-naïve	6 (0 – 29)
Binder-experienced	47 (0 – 204)

^a^See Table 2 for summary of types of previous phosphate binders used.

**Table 2.** History of phosphate binder use and pill burden.

Type of previous phosphate binder	Binder-experienced subgroup n = 71	
	n (%)	Mean pill count per day
Sevelamer^a^	37 (52)	11
Calcium^b^	20 (28)	9
Sevelamer + Calcium	10 (14)	16
Other	4 (6)	9

^a^Sevelamer carbonate is available in 800-mg tablets. ^b^Calcium acetate is available in 667-mg capsules.

**Table 3.** Mean (standard error of the mean) mineral bone biomarkers over time.

Month	Serum phosphorus (mg/dL)			Serum calcium (mg/dL)			iPTH (pg/mL)		
	N	Mean	SEM	N	Mean	SEM	N^a^	Mean	SEM
0 (Baseline)	92	6.55	0.17	92	9.00	0.08	81	480.5	46.4
1	92	5.90	0.18	92	8.97	0.08	56	469.6	46.0
2	90	5.74	0.16	90	8.96	0.08	63	477.5	44.5
3	90	5.75	0.15	90	9.03	0.08	56	466.3	41.4
4	89	5.63	0.16	89	9.07	0.06	69	475.2	45.0
5	86	5.38	0.15	87	8.99	0.08	58	502.0	46.3
6	81	5.41	0.17	82	9.08	0.08	59	488.0	43.0

^a^Laboratory values were collected per routine clinical care. iPTH was not typically measured on a monthly basis. iPTH = intact parathyroid hormone; SEM = standard error of the mean.
